# Analysis of ethics dumping and proposed solutions in the field of biomedical research in China

**DOI:** 10.3389/fphar.2023.1214590

**Published:** 2023-08-28

**Authors:** Bohua Liao, Yonghui Ma, Ruipeng Lei

**Affiliations:** ^1^ School of Philosophy, Huazhong University of Science and Technology, Wuhan, China; ^2^ School of Medcine, Xiamen University, Xiamen, China; ^3^ School of Marxism/Center for Ethics and Governance of Science and Technology, University of Electronic Science and Technology of China, Chengdu, China; ^4^ Center for Bioethics, Huazhong University of Science and Technology, Wuhan, China

**Keywords:** ethics dumping, biomedical research, ethics committee (EC), ethics governance, public policy

## Abstract

As international academic exchanges and cooperation deepen, China has actively engaged in international biomedical research collaboration and achieved significant success. However, these accomplishments have been accompanied by ethical controversies and issues, with ethics dumping being a recurrently discussed focus among scholars. This paper reviews ethics dumping incidents in China’s biomedical research field and analyzes the underlying causes to answer why China is often susceptible to ethics dumping. We argue that the primary reasons include weak ethical awareness among some researchers, an oversimplified research evaluation system, gaps in relevant ethics governance and oversight mechanisms, and limited capabilities of certain ethics committees. To address these issues, we propose five ethics governance recommendations: establishing refined ethics committees at various levels and types; advancing theoretical and practical research on science and technology ethics governance; strengthening legislation and regulation related to emerging science and technology; emphasizing self-regulation and capacity building of research institutions; and providing special protection and healthcare for victims of ethics dumping. The aim is to enhance China’s research supervision system and prevent similar ethics dumping incidents from recurring.

## 1 Introduction

Due to the close relationship between life sciences and human beings, discoveries and advancements in biomedical research often exhibit uncertainty and complexity in their impact on humanity. Emerging biotechnologies can be employed for the betterment of humankind, yet they can also be maliciously utilized to inflict harm. Following the CRISPR baby scandal occurrence in recent years, unethical conduct in biomedical research that contravenes research ethics and morality has increasingly garnered public attention. In academic and popular media discussions, a concept frequently associated with these incidents is “ethics dumping (Shih and Forsberg, 2023).”

## 2 The definition of ethics dumping

The term “ethics dumping” first emerged in the European Union’s Horizon 2020 research program in 2013 (Nordling, 2018). The European Commission defines ethics dumping as: “Due to the progressive globalization of research activities, the risk is higher that research with sensitive ethical issues is conducted by European organizations outside the EU in a way that would not be accepted in Europe from an ethical point of view. This exportation of these non-compliant research practices is called ethics dumping (Schroeder et al., 2018).” In the context of increasingly close international academic research exchanges, some researchers may inadvertently engage in ethics dumping by conducting research inappropriately in unfamiliar circumstances due to a lack of background knowledge and ethical awareness; However, there are also researchers who intentionally circumvent their home country’s stringent ethical regulations by transferring research explicitly prohibited in their home country to regions with less strict ethical regulations, consciously engaging in ethics dumping (Schroeder et al., 2019).

Due to economic development and historical factors, China’s ethical oversight of scientific research activities still lags behind that of Western developed countries, rendering China susceptible to ethics dumping. In recent years, incidents such as the Golden Rice Incident, the Berlin Heart case, the CRISPR baby scandal, and the body-to-head transplantation in China have exemplified instances of ethics dumping in the country’s biomedical research field.

## 3 Typical cases of ethics dumping in biomedical research in China

**The Golden Rice Incident** (Qiu, 2012; Enserink, 2014; Yang et al., 2014; Yu and Li, 2014): In June 2008, Chinese-American scholar Guangwen Tang from Tufts University smuggled approximately 1 kg of “Golden Rice” into China. Golden Rice refers to rice that has been genetically modified to contain the precursor of vitamin A, β-carotene, in its edible part, the endosperm. The carotene imparts a golden color to the rice, hence its name. For her study, Tang selected 72 students from a primary school in Jiangkou Town, Hengnan County, Hunan Province. Without obtaining informed consent from the subjects or their guardians, she deceived 72 children into consuming 60 g of Golden Rice for lunch every day for 21 days and tested their vitamin A levels. This conduct violated the fundamental ethical principle of respecting individuals in biomedical research. After the incident was exposed by Greenpeace in 2012, Guangwen Tang’s published papers were retracted, relevant domestic collaborative researchers were penalized, and each child involved in the study was ultimately compensated with 80,000 yuan.

**The Berlin Heart Case** (Ying, 2006a; Ying, 2006b; Ying, 2007; Linjuan, 2008; Liangjie and Xiaoe, 2013): In April 2004, 12-year-old Yiqing Zhou was admitted to Shanghai East Hospital to treat primary dilated cardiomyopathy. With the assistance of German-Chinese doctor Yuguo Weng (who did not possess surgical qualifications in China at the time), Zhou’s parents were deceived into allowing the illegal implantation of the “Berlin Heart”—an artificial heart product that had not obtained the medical device registration and permission in China—into Zhou’s body and the illegal performance of so-called “stem cell treatment.” Zhou eventually passed away 15 months later due to secondary multi-organ failure. The study deliberately blurred the distinction between “clinical treatment” and “clinical trial,” extracting Zhou’s cerebrospinal fluid and live skeletal muscle cells for stem cell culture experiments under the guise of treatment. The trial project was jointly conducted by Shanghai East Hospital and Yale University in the United States, with the hospital providing cell samples and Yale University’s cell research institution providing stem cell culture equipment. At the time, stem cell therapy was still in the animal experimentation stage worldwide. The so-called “cultured stem cells” were extremely immature, resulting in the death or disability of several Chinese patients who underwent similar trials, including Zhou.

One of China’s most influential ethics dumping cases is **the CRISPR baby scandal** (Cohen, 2019; Cyranoski, 2019; Greely, 2019): In November 2018, Jiankui He used self-raised funds, forged ethical review documents, and arranged for others to take blood tests in subjetcts’ place to evade supervision deliberately. He illegally employed the CRISPR technology to modify the genes of fertilized eggs, intentionally violating the National Health Commission of China’s (NHC) ban on embryonic research and ultimately resulting in the birth of the world’s first three gene-edited babies. Before commencing the experiment, Jiankui He had extensive discussions with several closely related American scientists. These scientists were fully aware of the international consensus and guidelines on embryonic research yet allowed the entire incident to happen. When Jiankui He explained the trial to participating couples, his graduate supervisor, Professor Michael Deem of Rice University in the United States, was present; Jiankui He’s postdoctoral supervisor Stephen Quake was aware of it but did not intervene; and Jiankui He’s company’s technical consultant and Nobel laureate Craig Mello stated after learning of it: “I’m glad for you, but I’d rather not be kept in the loop on this.”

China has also had successful instances of preventing ethics dumping. One such case is **body-to-head transplantation (BHT)** (Furr et al., 2017; Wolpe, 2017; Ruipeng and Peng, 2019): In 2018, Dr. Xiaoping Ren of Harbin Medical University and his collaborator Sergio Canavero initiated a research project to implement head transplantation, or more precisely, BHT. According to Canavero’s plan, the donor and patient’s heads would first be cooled to 12–15°C before a highly sharp blade was used to sever both heads and spinal cords. The two ends of the spinal cord would then be fused together with polyethylene glycol to promote cell fusion. After the muscles and blood supply were successfully connected, the patient would be placed in a coma for 1 month to limit movement of the newly connected neck, while electric shocks were used to stimulate the spinal cord and strengthen its new connection. After about 1 month of coma, the patient would be able to move, have sensation in their face, and speak with a voice. Notably, Canavero originally submitted this research plan to his university in Turin but was fired instead of having his research plan approved. Aware that no country in Europe or North America would approve this research plan, Canavero came to China and found a partner in Dr. Xiaoping Ren at Harbin Medical University. Based on Xiaoping Ren’s prior experience with primate head transplantation experiments, the two collaborated on a BHT between two corpses and reported successful surgery in relevant media. Due to the complex ethical, legal, and social issues raised by BHT, it attracted significant attention from medical, ethical, and legal communities in China and internationally, eliciting criticism, questioning, and condemnation. Ultimately, the NHC halted the world’s first clinical trial of BHT, planned to be conducted in China.

## 4 Analysis of the causes of ethics dumping in biomedical research in China

### 4.1 Some researchers exhibit a deficiency in ethical awareness

The fundamental cause of ethics dumping is the weak or absent ethical awareness of some scientific researchers. Some researchers in China lack knowledge of research and medical ethics, leading to disregard for ethical issues in their research. Even a few researchers mistakenly believe that “there are no forbidden areas in scientific research” and that “overemphasis on ethics is an obstacle to scientific research (Dongping, 2009).” There is also the so-called “overtaking on a curve”—the notion that China has fewer ethical restrictions than the West, allowing for more uninhibited research and better and faster capture of outstanding achievements (Junjun, 2009). This misunderstanding of scientific ethics often leads researchers to pursue being “ahead of the world” or “the first in the world” in their research practice. It also leads some international collaborators to deliberately seek loopholes and vacancies in relevant laws and regulations, conducting research in China to circumvent the “obstacles” of their home country’s system.

### 4.2 The evaluation system is overly simplistic

In the field of biomedical research, there is a saying “publish or perish,” referring to the pressure on researchers to publish academic papers in order to succeed in their academic careers (Miller et al., 2011). While it is undeniable that the number of high-level academic papers published is an important indicator of a researcher’s academic ability, however, in practice, this often becomes the “only” indicator. A researcher’s academic status, funding received, and promotion of positions and duties are often directly linked to the quality and quantity of published papers. The causes of this overly simplistic evaluation system can be analyzed from two perspectives: national science and technology strategy and the considerations of individual scholars.

On the one hand, China’s modern history of invasion and humiliation by industrialized foreign powers has fostered a tradition of thought that science and technology can save the nation and serve as sources of prosperity. (Wu, 2019). This thought has deeply influenced the development of China’s science and technology strategy and policies. China has consistently viewed the advancement of science and technology as the key to achieving economic growth and enhancing national strength. To achieve this goal, it often attracts and motivates scientific researchers by offering competitive salaries to top scientists, funding for scientists wishing to establish laboratories in China, and special funds to support international scientific cooperation (Salter, 2009). While these policies have enabled China to make significant progress in science and technology, they have also led a small number of researchers to disregard research ethics in pursuit of results at any cost. The aforementioned concepts of “there are no forbidden areas in scientific research” and “overtaking on a curve” are specific manifestations of this phenomenon.

On the other hand, the quality of papers is often reflected in the number of times they are cited. Novel and unconventional research results can usually attract more attention from academic peers and thus receive more citations. Some researchers choose to engage in radical scientific research through ethics dumping for their own benefit, hoping to produce sensational research results that will attract attention from the outside world and investors. Such researchers can be found in both developed and developing countries, including Jiankui He from China (Krimsky, 2019), Hwang Woo Suk from South Korea (Gottweis and Triendl, 2006), Geeta Shroff from India (Zhang and Burton, 2022), and Paolo Macchiarini from Sweden (Berggren and Karabag, 2019), etc.

### 4.3 There are loopholes in the relevant ethics governance and supervision mechanisms

Due to the relatively low level of scientific and technological development and disciplinary establishment, as well as the small size of research teams in interdisciplinary fields such as bioethics and biomedical ethics, there are inherent weaknesses in China’s ethics governance and oversight mechanisms. Taking the CRISPR baby scandal as an example, both the Southern University of Science and Technology and the hospital that approved Jiankui He’s research lacked effective ethical review prior to the start of clinical trials and corresponding supervision throughout the entire process (Yan et al., 2021). Although Jiankui He’s actions violated many Chinese regulations and guidelines, including (Wang et al., 2019; Peng et al., 2020): *Ethical Guidelines for Human Embryonic Stem Cell Research* (2003), *the Ethics Principles for Human Assisted Reproductive Technology and Human Sperm Bank* (2003), *Measures for Ethical Review of Life Science and Biomedical Research Involving Humans* (2016), and *the Safety Management Measures for Biotechnology Research and Development* (2017), the fact that these regulations and guidelines were merely departmental rules or normative documents lacked or even without legally binding, corresponding accountability, and punitive measures. Under the conditions at that time, it was difficult to hold Jiankui He accountable for his actions based on these regulations or guidelines (Zhai et al., 2019).

Moreover, due to the controversy surrounding the moral and legal status of human embryos, there are varying interpretations of their status among different laws and regulations in China before the CRISPR baby scandal (Jiang and Rosemann, 2019). This uncertainty has resulted in a multi-level regulatory system that has caused confusion and created a regulatory vacuum, which has provided opportunities for irresponsible researchers to exploit these loopholes for ethics dumping. While some scholars have cited the case of Junjiu Huang as evidence that China’s ethical regulatory system is similar to that of Western countries (Zhai et al., 2016), it is important to note that Huang’s research differs fundamentally from that of Jiankui He. Huang used tri-pronuclear zygotes within 14 days that could not develop into humans, whereas He directly edited human reproductive cells (Lei and Qiu, 2020; Zhang et al., 2022). Therefore, in response to the CRISPR baby scandal, China promulgated and revised relevant laws and regulations involving human embryos, which mainly include *the Biosecurity Law*, *Basic Healthcare law*, *Regulations on the Management of Human Genetic Resources*, and so on, and established a National Science and Technology Ethics Committee to improve the regulatory system at both the legislative and administrative levels (Song and Joly, 2021a).

### 4.4 Some ethics committees exhibit inadequacies in their review capabilities

The NHC promulgated and revised the “Measures for Ethical Review of Life Science and Biomedical Research Involving Humans” in 2010, 2016, 2020, and 2023 respectively (Normile, 2023), requiring institutions conducting drug trials and clinical research involving humans to establish ethics committees. However, in practice, certain institutions—particularly grassroots and private medical institutions—have exhibited deficiencies in review capabilities, superficial review processes, and a lack of independence among their ethics committees (Ruipeng and Yi, 2022). These issues arise from several factors: inadequate professional expertise among committee members impedes their ability to conduct thorough ethical reviews; the relatively marginalized position of ethics committees within some institutions leaves them vulnerable to pressure from researchers and other internal or external forces that can compromise their independent judgment; and the susceptibility of private medical organizations’ ethics committees to pressure from investors and market forces further hinders their ability to conduct effective ethical reviews.

## 5 Policy recommendations

### 5.1 Refining the establishment of science and technology ethics committees at different levels and types

China has established a National Science and Technology Ethics Committee with sub-committees responsible for life sciences, medicine, and artificial intelligence (Ru and Huina, 2023). However, each sub-committee encompasses an extensive range of scientific research and application domains, resulting in a considerable workload for top-level institutional design and the development of ethical guidelines. To address this challenge, China could emulate the working mechanisms of national-level ethics committees and government agency ethics committees in Europe and America by establishing working groups within its sub-committees. These working groups would be organized according to specific emerging science and technology fields and tasked with drafting, writing, and periodically revising ethics governance norms and guidelines for their respective domains.

Provinces and cities should expedite the establishment of regional (provincial and municipal) science and technology ethics committees to create a comprehensive, well-regulated, and harmonized system for the governance of science and technology ethics. These regional committees would be responsible for the training, assessment, and capacity building of various institutions’ ethics committees within their jurisdiction. Regional committees should also regularly evaluate relevant committees’ operations and establish effective accountability mechanisms.

Additionally, a graded ethical review and approval mechanism could be implemented to provide full-process oversight of clinical trials, high-risk research activities in science and technology ethics that may pose significant ethical concerns, as well as related international collaborative research endeavors. It can draw upon the practices employed in Japan. Japan’s scientific research ethics governance system is primarily led by the government and research institutions, operating in parallel with central and subordinate institutions (Danyang and Wenxia, 2016). Its administrative structure and institutional arrangements bear similarities to those in China. Specifically, in Japan, there are two primary methods of ethical review: one requires only that the Institutional Review Board (IRB) review the research plan, while the other is a “two-step review” process, whereby high-risk research plans involving human embryo research, gene editing, and the like must be reviewed by the IRB and confirmed by the national government (Aikyo et al., 2023). China should promptly establish a high-risk research list in science and technology and dynamically adjust it as circumstances dictate. The research included on the list should be reviewed by regional ethics committees and submitted to the National Science and Technology Ethics Committee for confirmation upon approval.

### 5.2 Furthering theoretical and practical research on the ethics governance of science and technology

Since the dawn of the 21st century, global scientific innovation has entered an unprecedented period of intense activity. Emerging technologies in this century are characterized by significant uncertainty and dual-use potential and may give rise to novel ethical challenges (Meng and Wang, 2023). To advance research on science and technology ethics governance in this new era—in line with national requirements for the development of science and technology ethics—it is imperative to promote the establishment of disciplinary systems and academic frameworks for science and technology ethics governance research in China. To this end, China should establish several national-level science and technology ethics governance research institutions at the earliest opportunity. These institutions would be selected from among qualified universities and research organizations across the country to form a network of sustainable professional think tanks that can support the development of national science and technology ethics governance systems through policy consultation. Institutionally, cross-disciplinary research on science and technology ethics governance should be assigned a clear disciplinary affiliation. At the undergraduate level, relevant courses on the science and technology ethics governance can be offered in the disciplines of applied ethics, law, political science, etc. At the graduate level, students interested in this new interdisciplinary field can be recruited in related disciplines to lay a sustainable foundation for talent cultivation, interdisciplinary construction, as well as the development of academic frameworks and regulatory systems for science and technology ethics governance.

### 5.3 Consolidating legislative and regulatory frameworks for the ethics governance of emerging science and technology

While *the Biosecurity Law*—which forms the core of China’s national biosecurity legal system—has been enacted, and a legal framework for human genetic resources is under development, efforts to improve protection and supervision systems for human subjects in biomedical research and clinical trials of new biomedical technologies are ongoing (Peng et al., 2022; Bohua et al., 2023). However, disagreements remain over these regulatory systems’ conceptualization, content, structure, and implementation. Notably, relevant subordinate laws and supporting implementation rules and enforcement measures have yet to be introduced. This presents challenges for governance and oversight.

The state should delineate the supervisory responsibilities of various departments under *the Biosecurity Law* and specify measures for implementing biosecurity systems and graded, classified management. Coordination with relevant laws and regulations—including *the Civil Code, Criminal Law, Personal Information Protection Law, Administrative License Law, Administrative Punishment Law, Law of Scientific and Technological Progress*, etc.,.— must also be considered. Appropriate administrative, civil, and criminal penalties should be established to ensure that there is a legal basis for addressing serious ethical incidents (Song and Joly, 2021b). Within the civil domain, corresponding relief and care measures should also be introduced to enable relevant parties (such as subjects in cases of ethics dumping) and stakeholders to protect their legitimate rights and interests through legal means when these are infringed upon.

### 5.4 Emphasizing self-regulation and capacity building among relevant research institutions

Most scientific researchers in China are responsible individuals with a strong awareness of safety risks. However, given the rapid pace of scientific advancement, institutional oversight inevitably lags behind. As such, the self-discipline and self-governance of the scientific community are essential and warrant encouragement and support (Lei and Qiu, 2020). Efforts should be made to move away from a research culture prioritizing publications above all else and dismantle short-sighted evaluation systems that reward quick success and instant gratification. Instead, basic research should be vigorously supported, and the role of academic and social organizations in promoting self-discipline should be recognized.

Besides, qualified instructors should be engaged to deliver science and technology ethics courses across various levels and categories, emphasizing teacher training and the development of course materials and textbooks. Education is a long-term undertaking and forms the bedrock of China’s science and technology ethics governance system. As such, Sustainable education, training, and capacity-building efforts should be implemented to establish a comprehensive capacity-building framework for science and technology ethics governance. Regular institutionalized education and training should be provided to front-line scientific researchers, ethics committee members, administrative managers, industry personnel, and media practitioners to facilitate the internalization and normalization of self-education and self-regulation (Lei et al., 2019).

The advancement of science and technology plays a pivotal role in accelerating the growth of China’s economy and augmenting its comprehensive national power. Ethical considerations in science and technology serve to guide and protect its development. Progress in these fields is inextricably linked to critical reflection by scholars in the humanities. It is imperative that we foster meaningful interdisciplinary collaboration and deep academic exchange between scientists and scholars in the humanities and social sciences (Fu and Nielsen, 2023). By collectively reviewing and learning from successful practices in science and technology ethics governance in the West, we can develop and implement our own governance programs embedded in the Chinese social context and tailored to China’s circumstances.

### 5.5 Providing special protective and supportive measures for subjects of ethics dumping incidents

As the three gene-edited babies grow older and Jiankui He is released from prison, international attention to this incident—particularly with regard to the children—continues to intensify (Lei and Qiu, 2022). It is recommended that China follow the example set by the United Kingdom in establishing the Warnock Committee after the world’s first test-tube baby sparked significant ethical controversy (Warnock, 1985). A state-led expert working group could be convened to explore feasible protective and supportive measures. The UK’s approach not only defused a crisis and facilitated the orderly development of assisted reproductive technology norms but also safeguarded the first test-tube baby’s health interests and social rights. Furthermore, it established international consensus on science and technology ethics, such as the “14-day standard” for embryonic research, serving as a model for balancing scientific innovation with ethical norms. It is anticipated that a similar working mechanism established by China could likewise become an international exemplar for the ethical governance of reproductive gene editing, enabling China to contribute its perspective to global science and technology governance while assuming a leadership role.

## Data Availability

The original contributions presented in the study are included in the article/Supplementary material, further inquiries can be directed to the corresponding author.
